# An exploratory study evaluated the 30 most commonly reported medications in the United States food and drug administration’s adverse event reporting system that are associated with the occurrence of kidney stones

**DOI:** 10.3389/fphar.2024.1377679

**Published:** 2024-12-18

**Authors:** Erhao Bao, Yang Yang, Binglei Jiang, Ben Wang, Ying Liu, Lin Yang, Long Xia, Pingyu Zhu

**Affiliations:** ^1^ Urology Department, Sichuan Provincial People’s Hospital East Sichuan Hospital and Dazhou First People’s Hospital, Dazhou, China; ^2^ Department of Urology, Affiliated Hospital of North Sichuan Medical College, Nanchong, Sichuan, China; ^3^ Traditional Chinese Medicine department, Sichuan Provincial People’s Hospital East Sichuan Hospital and Dazhou First People’s Hospital, Dazhou, China; ^4^ Department of Ultrasound, Affiliated Hospital of North Sichuan Medical College, Nanchong, Sichuan, China

**Keywords:** exploratory study, medications, FDA, kidney stones, FAERS

## Abstract

**Objective:**

This research project aimed to identify and analyze the top 30 drugs most commonly associated with kidney stone formation using data from the U.S. Food and Drug Administration’s Adverse Event Reporting System (FAERS) database. The study will focus on determining the reported Odds ratio (ROR) and Proportional Report ratio (PRR) of each identified drug to assess its potential risk of contributing to the development of kidney stones in order to effectively manage and reduce the overall burden of kidney stones worldwide.

**Background:**

Kidney stones are a common urinary system disease, and their formation is influenced by multiple factors, including medications, metabolic syndrome, environmental factors, genetic susceptibility, urinary tract abnormalities, and obstructive pathology. Among these factors, medications are an important and clearly defined cause of kidney stone formation. Currently, drug-induced kidney stones account for 1%–2% of all cases of kidney stones. Although the proportion may appears modest, its contribution to the overall prevalence of kidney stones should not be overlooked. The high incidence and frequent recurrence of kidney stones pose significant challenges to prevention efforts. Therefore, a thorough understanding of which medications may trigger stone formation is crucial for effectively managing and reducing the burden of this global health issue.

**Method:**

This study utilized the FDA Adverse Event Reporting System (FAERS) database maintained by the U.S. Food and Drug Administration to extract raw data from 1 January 2010, to 31 March 2024. Duplicate data was removed following the FDA-recommended method. Subsequently, data related to drug-induced kidney stones was extracted by linking the DEMO table, DRUG table, and REAC table using specific primaryid numbers, resulting in a total of 37,781 drug records and 37,027 demographic records. The data was sorted based on the frequency of drug-induced kidney stones, and the top 30 drugs associated with kidney stone formation were selected for analysis. The primary analytical methods employed were disproportionality analyses, calculating the Reporting Odds Ratio (ROR) with its 95% Confidence Interval (CI) and Proportional Reporting Ratio (PRR) to reveal the association between drugs and adverse kidney stone events.

**Results:**

We ranked the 30 most common drugs based on their ROR and PRR values. The three drugs most commonly associated with kidney stones were Atazanavir (ROR 46.35, 95% CI 43–50, PRR 44.9), Topamax (ROR 19.44, 95% CI 17.66–21.40, PRR 19.19), and Prevacid (ROR 12.67, 95% CI 11.62–13.82, PRR 12.57). Drug categories most commonly associated with kidney stones were antiretroviral drugs, proton pump inhibitors, and antiepileptic drugs.

**Conclusion:**

Our research has summarized a list of potential drug categories associated with kidney stones. Clear understanding of the risk and frequency of drug-induced kidney stones caused by specific medications can reduce the likelihood of patients developing the condition. Clinical doctors should keep vigilant during diagnosis and treatment processes, and communicate relevant risk information to patients.

## 1 Introduction

Renal calculi, a prevalent affliction of the urinary system, are now universally acknowledged by experts worldwide to be multifactorial in etiology, stemming not from a singular cause but rather from a confluence of pharmaceuticals, metabolic syndrome, environmental factors, genetic predispositions, hydration levels, urinary tract anomalies, and obstructive pathologies (Ferraro et al., 2020; Daudon et al., 2018a; Karoli et al., 2021).Specifically, pharmaceuticals have been identified as a significant contributor to the formation of kidney stones. To date, drug-induced kidney stones account for 1%–2% of all kidney stones (Daudon et al., 2018b).Based on the mechanisms of stone formation, the implicated drugs can be classified into two categories. The first category includes insoluble medications that promote crystal and stone formation. Examples include protease inhibitors such as azanavir used in the treatment of human immunodeficiency virus (HIV) and sulfadiazine used for treating toxoplasmosis. The second category includes medications that facilitate stone formation through their metabolic effects, such as carbonic anhydrase inhibitors like acetazolamide or topiramate (Sighinolfi et al., 2019).

Throughout history, sulfonamide drugs were among the first medications implicated in the formation of kidney stones. Shortly after their introduction to human use, studies revealed associations between sulfonamide drugs and the occurrence of kidney stones and acute renal failure. Numerous reports have documented the links between sulfonamide drugs and renal diseases (Antopol and Robinson, 1939; Barnes and Kawaichi, 1943; Abaza, 1946; Lucas et al., 1982; Lehr, 1957). However, in the ensuing decades, there were only sporadic observations of drug-induced kidney stone formation in the scientific literature. It was not until the early 1980s that the concept of drug-induced kidney stones began to receive attention. In 1980, Ettinger and colleagues conducted an analysis of around 50,000 kidney stone patients and found that 181 of them had been exposed to triamterene, with an incidence rate of drug-induced kidney stones at 0.4% (Ettinger et al., 1980).During 1982 to 2002, studies conducted in Switzerland, Spain, and France reported varying rates of urinary tract stone formation related to different medications, with triamterene remaining the most common substance implicated. The incidence rates were 0.4% in Switzerland and Spain, while in French studies, the overall occurrence rate of drug-related stones was 1% (R´eveillaud and Daudon, 1986; Rapado et al., 1987a; Daudon et al., 1982; Moesch et al., 1989).Subsequently, other drug components such as indinavir were also identified in kidney stones.

Although the associations between kidney stones and certain medications is often mentioned, there is a lack of large-scale studies in the literature to specifically establish which class of drugs is most likely to cause kidney stones. There are two main reasons as follows: firstly, the concept of drug-induced kidney stones was not introduced until the early 1980s and received attention relatively late. Secondly, researches on drug-induced kidney stones have mostly focused on a specific class of drugs, with only sporadic reports, making it a huge challenge to systematically verify whether commercially available drugs can cause kidney stones. Currently, there is no systematic list recommending potential drugs that may cause kidney stones. Therefore, it is necessary to identify and analyze the drugs most likely to cause kidney stones through a large amount of adverse drug reaction data.

Our research aims to address this issue by evaluating the United States Food and Drug Administration Adverse Event Reporting System (FAERS). FAERS is a drug safety monitoring database used to report real-world adverse events (AE). We identified the drugs most relevant to “kidney stones” in the FAERS database and identified the top 30 drugs most likely to cause kidney stones. We classified the drugs and used the Reporting Odds Ratio (ROR) and Proportional Reporting Ratio (PRR) to depict the extent to which each drug affects kidney stones. The higher the ROR and PRR values, the greater the likelihood that the drug causes kidney stones. We also analyzed the relationships between drugs and kidney stones and discussed the research findings. Ultimately, we compiled a list of the 30 most common drugs that lead to kidney stones. This list can be used by physicians to select treatment options for kidney stone patients, assess the potential risk factors for kidney stone formation, effectively manage and reduce the global burden of kidney stones. Additionally, it can serve as a reference for future epidemiological studies on drug-induced kidney stones or reveal potential mechanisms of kidney stone formation.

## 2 Data and methods

### 2.1 Data source

FAERS is an open database maintained by the FDA, collecting adverse event and medication error reports. It aids in the post-marketing safety oversight of drugs and therapeutic biological products. Users can access the database for free (https://fis.fda.gov/extensions/FPD-QDE-FAERS/FPD-QDE-FAERS.html). The database includes details such as patient demographics, drug information, reaction details, patient outcomes, and report origins. Users can download all tables from the official website and merge and summarize data quarterly from 2010 Q1 to 2024 Q1, categorized under patient demographics, drug information, adverse event details, patient outcomes, report sources, drug therapy dates, and drug indications.

### 2.2 Data deduplication

The FAERS database, characterized by spontaneous reporting, contains duplicate reports or deleted/removed reports. To address these issues, the FDA provides guidelines for data de-duplication and a list of reports that should be excluded. This study adhered strictly to the data cleaning guidelines available on the FDA website.

According to the FDA’s recommended method for data de-duplication, the primary identifiers - PRIMARYID, CASEID, and FDA DT - from the DEMO table were used. Reports with the same CASEID were retained based on the highest FDA DT value. Additionally, among reports with the same CASEID and FDA DT, the report with the highest PRIMARYID value was selected. These principles were applied in R to de-duplicate the original data. We get the data of the DEMO table after deduplication is 17,379,609.
In the FAERS database, the CASEID serves as a unique identifier for each report. Reports with the same CASEID could represent different versions of the same report or observations by multiple observers. Therefore, de-duplication is necessary for reports with the same CASEID. In the FAERS database, de-duplication typically involves retaining the report with the highest FDA DT value. FDA DT represents the submission date of the report, and later reports typically overwrite earlier ones. By retaining the report with the highest FDA DT value, the latest report is ensured, thereby avoiding duplication and misinterpretation of the data.

### 2.3 Standardization of drug names

The DiAna dictionary, an open-source tool in the drug vigilance community, serves as a standardized reference for drug names in the FAERS database (Fusaroli et al., 2024). We adopted the methodology used by the DiAna dictionary for standardizing drug names, which involved searching for all unique terms in the Prod_ai and Drugname fields, converting them to lowercase, uppercase, removing multiple spaces, leading and trailing spaces, and punctuation, and spaces between parentheses. We then merged these pre-formatted unique terms with the brand names and ingredients recorded in RxNorm (https://www.nlm.nih.gov/research/umls/rxnorm/) and the WHO-ATC (ATC/DDD, 2023) classification to create an initial dictionary. This dictionary was used to translate all brand names in the data collection/parsing dataset into their generic equivalents.

The process of standardizing drug names is crucial for accurate analysis and comparison of adverse event reports. By converting brand names into their generic equivalents, researchers can identify patterns and trends across different drugs that may share the same active ingredient or therapeutic class. This standardization also helps in reducing the variability in reporting and improves the comparability of data across different studies and analyses.

### 2.4 Data mining

**FIGURE 1:**
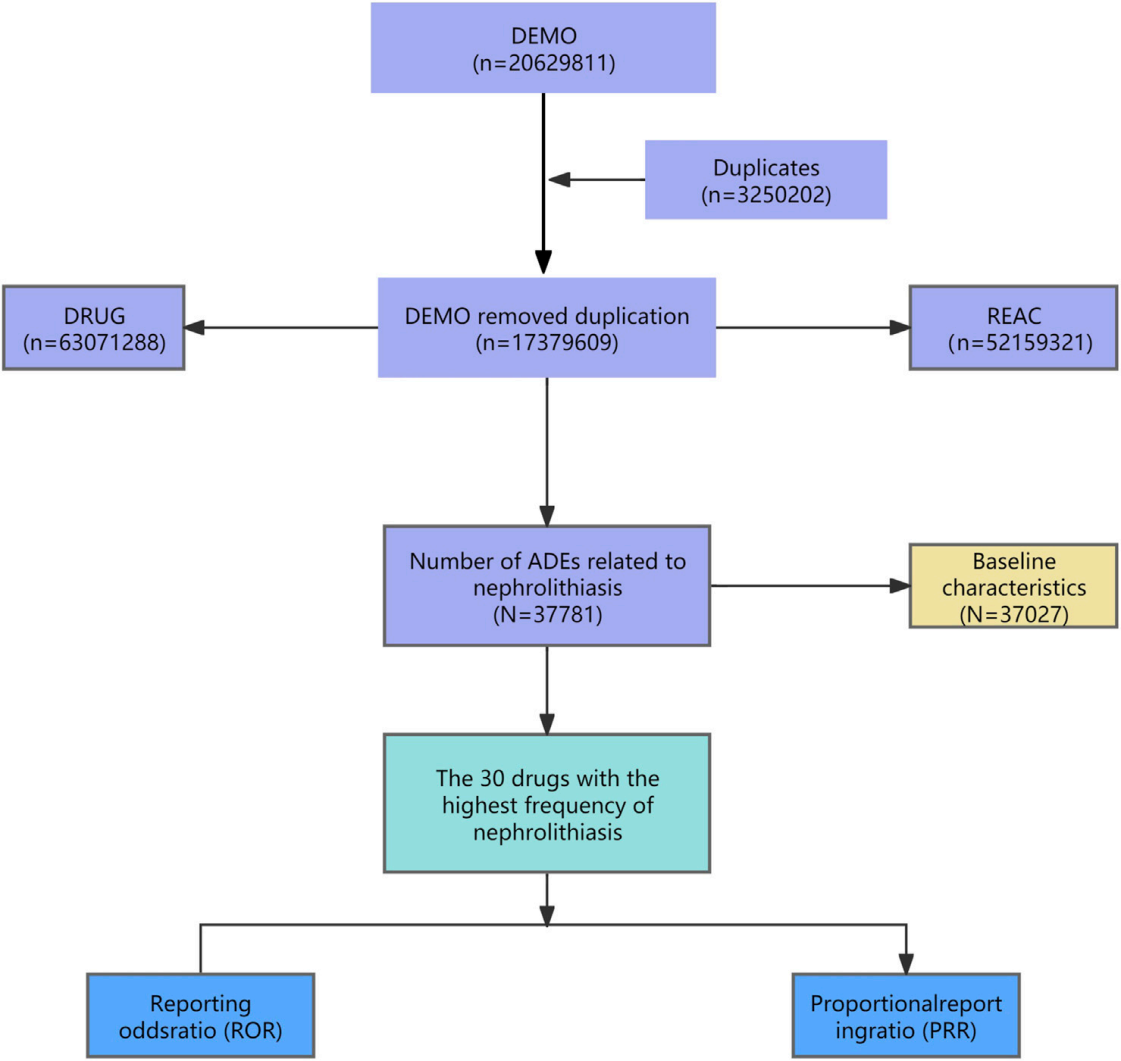
Flowchart.

We first extracted the data of 1 January 2010, to 31 March 2024, and obtained the DEMO table after deduplication (n = 17,379,609). At the same time, the DEMO table was associated with DRUG table and REAC table respectively by specific primaryid number, and adverse reactions caused by drugs were set as nephrolithiasis for extraction. A total of 37,781 drug data causing kidney stones were obtained, and 37,027 basic demographic information were obtained. The drugs were sorted according to the occurrence frequency (a) value, and the top 30 drugs were analyzed. The above processes were carried out in R language (4.3.2), and the relevant data were processed in the flow chart (Figure 1).

### 2.5 Data analysis

The main analytical method used in this study was the disproportionality method. Specifically, we calculated the Reporting Odds Ratio (ROR) and its 95% confidence interval (CI), as well as the Proportional Reporting Ratio (PRR). These methods were based on the parameters of the 2 × 2 table for signal detection, as shown in Table 1. The algorithms and standards for these methods are outlined in Table 2. A higher ROR value and PRR value indicate a stronger signal, suggesting a greater correlation between the target drug and the target adverse event (AE). The rules for generating signals were as follows: (1) the lower limit of the 95% CI > 1; (2) the number of reports a ≥3 (Tian et al., 2022).

**TABLE 1:** 2 × 2 table.

Database	Target AE (nephrolithiasis)	All other AE	Total
Target drug	a	b	a+b
All other drugs	c	d	c+d
Total	a+c	b+d	a+b+c+d

a, number of reports containing both the target drug and nephrolithiasis; b, number of reports containing other AE, of the target drug; c, number of reports containing the nephrolithiasis of other drugs; d, number of reports containing other drugs and other AE.

**TABLE 2:** Formulas and threshold values of ROR and PRR.

Method	Formulas	Inclusion criteria for AE
ROR	$\text{ROR}=\frac{\left(a/c\right)}{\left(b/d\right)}$	95%CI≻1 a≥3
	$\text{SE}\left(\ln R0R\right)=\sqrt{\frac{1}{a}+\frac{1}{b}+\frac{1}{c}+\frac{1}{d}}$	
	$95\%\text{CI}=e^{\ln\;\left(R0R\right)\pm 1.96\sqrt{\frac{1}{a}+\frac{1}{b}+\frac{1}{c}+\frac{1}{d}}}$	
PRR	$\text{PRR}=\frac{a/\left(a+b\right)}{c/\left(c+d\right)}$	95%CI≻1 a≥3
	$\text{SE}\left(\ln PRR\right)=\sqrt{\frac{1}{a}-\frac{1}{a+b}+\frac{1}{c}-\frac{1}{c+d}}$	
	$95\%\text{CI}=e^{\ln\;\left(PRR\right)\pm 1.96\sqrt{\frac{1}{a}-\frac{1}{a+b}+\frac{1}{c}-\frac{1}{c+d}}}$	

### 2.6 Subgroup analysis of drug categories

First, we categorize the first 30 drugs, with the top three categories being anti-rheumatic drugs, parathyroid hormone-related drugs, and antiviral drugs. We have conducted subgroup analysis on these top three drug classes, using the disproportionality method as the primary analytical approach. For more detailed analytical methods, please refer to Tables 1, 2. Subsequently, we compare the drug categories differentially, with the differences in the meanings of a, b, c, and d as outlined in Table 3. We then calculate the Reporting Odds Ratio (ROR) and its 95% confidence interval (CI) using a 2 × 2 contingency table, based on the disproportionality method applied to drug category differential comparison. The calculation formula is: $\text{ROR}=\frac{\left(a/c\right)}{\left(b/d\right)},95\%\text{CI}=e^{\ln\;\left(R0R\right)\pm 1.96\sqrt{\frac{1}{a}+\frac{1}{b}+\frac{1}{c}+\frac{1}{d}}}$ The signal detection criteria are as follows: (1) the number of reports (a) ≥ 3; and (2) ROR >1, with the lower limit of the 95% CI > 1, indicating that drug class A is more likely to be associated with the occurrence of kidney stones compared to drug class B, with a larger ROR indicating a stronger association (Yang and Chen, 2024).

**TABLE 3:** 2 × 2 table (based on the disproportionality method applied to drug category differential comparison).

Database	Target AE (nephrolithiasis)	All other AE	Total
drug class A	a	b	a+b
drug class B	c	d	c+d
Total	a+c	b+d	a+b+c+d

(a) represents the number of reports of kidney stones as an adverse event associated with drug category A.

(b) represents the number of reports of any adverse event other than kidney stones associated with drug category A.

(c) represents the number of reports of kidney stones as an adverse event associated with drug category B.

(d) represents the number of reports of any adverse event other than kidney stones associated with drug category B.

## 3 Results

**FIGURE 2:**
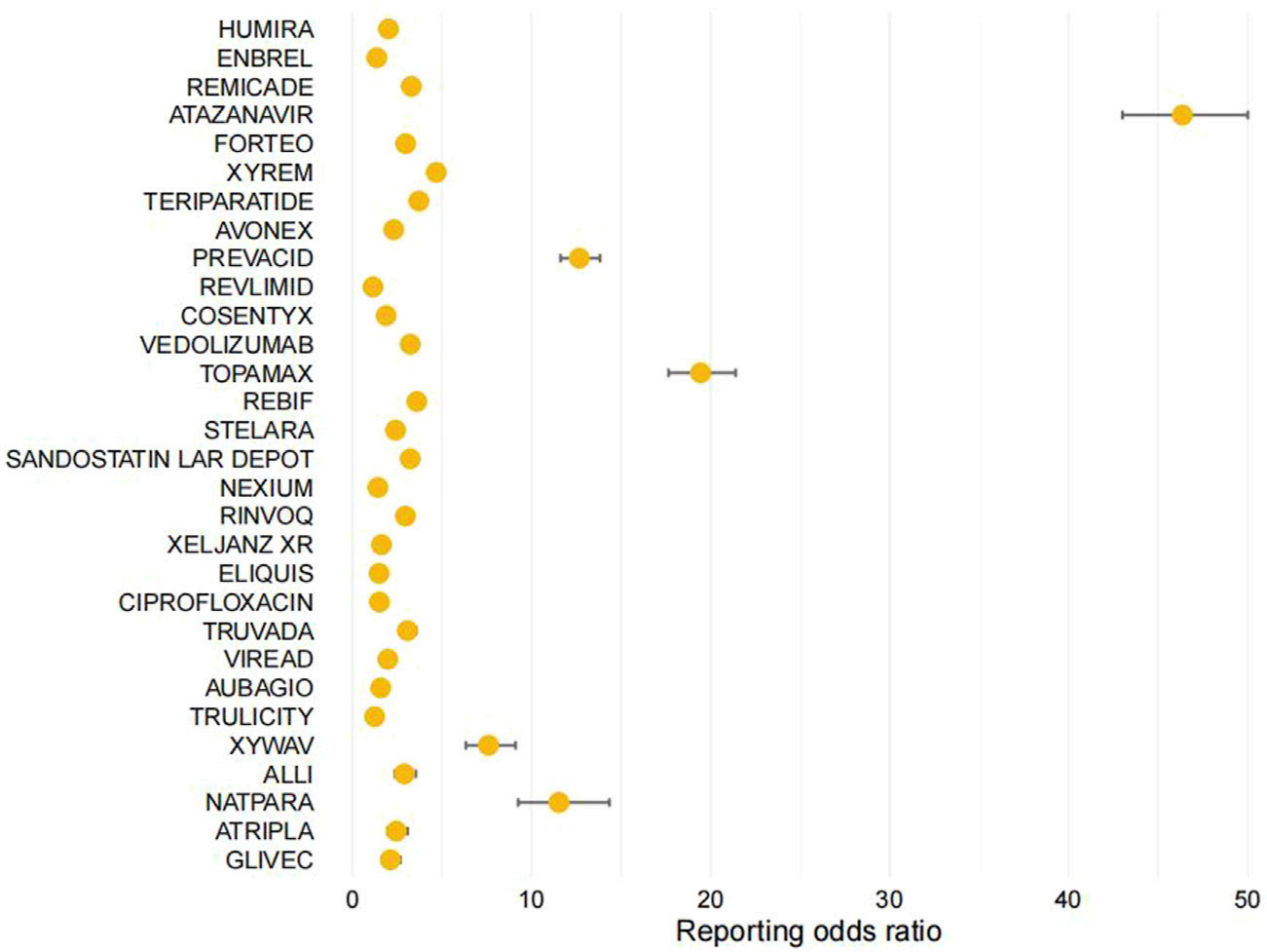
Forest Plot: Analysis of Reporting Odds Ratio (ROR) of the Top 30 Most Common Medications Associated with Kidney Stone Formation in the FDA's FAERS Database.

We extracted a total of 37,781 entries containing the term “nephrolithiasis.” Considering further evaluation of the top 30 drugs with the highest frequency of appearance in the reports, the range was from 73 to 2,429. The ROR values for this group of drugs range from 1.15 to 46.35, as shown in Table 4 and Figure 2. We have identified the three most relevant drugs associated with the occurrence of kidney stones. These drugs are Atazanavir (Reporting Odds Ratio (ROR) of 46.35, 95% Confidence Interval (CI) of 43–50, Proportional Reporting Ratio (PRR) of 44.9), Topamax (ROR of 19.44, 95% CI of 17.66–21.40, PRR of 19.19), and Prevacid (ROR of 12.67, 95% CI of 11.62–13.82, PRR of 12.57). Additionally, the top three drugs with the highest number of reported cases are Humira (2,429 cases), Enbrel (1,279 cases), and Remicade (794 cases).

**TABLE 4:** Analysis of reporting odds ratio (ROR) and proportional reporting ratio (PRR) of the top 30 most common medications associated with kidney stone formation in the FDA’s FAERS database from 1 January 2010, to 31 March 2024.

Ranking	Medication	a	b	c	d	ROR	ROR lower bound, 95% CI	ROR upper bound, 95% CI	PRR
1	HUMIRA	2,429	1,722,659	35,352	50,398,881	2.01	1.93	2.09	2.01
2	ENBREL	1,279	1,312,960	36,502	50,808,580	1.36	1.28	1.43	1.36
3	REMICADE	794	338,134	36,987	51,783,406	3.29	3.06	3.53	3.28
4	ATAZANAVIR	702	21,282	37,079	52,100,258	46.35	43	50	44.9
5	FORTEO	697	327,873	37,084	51,793,667	2.97	2.75	3.2	2.96
6	XYREM	686	205,027	37,095	51,916,513	4.68	4.34	5.05	4.67
7	TERIPARATIDE	589	221,807	37,192	51,899,733	3.71	3.42	4.02	3.7
8	AVONEX	557	335,159	37,224	51,786,381	2.31	2.13	2.51	2.31
9	PREVACID	525	57,903	37,256	52,063,637	12.67	11.62	13.82	12.57
10	REVLIMID	524	627,869	37,257	51,493,671	1.15	1.06	1.26	1.15
11	COSENTYX	523	385,961	37,258	51,735,579	1.88	1.73	2.05	1.88
12	VEDOLIZUMAB	446	191,855	37,335	51,929,685	3.23	2.94	3.55	3.23
13	TOPAMAX	425	30,482	37,356	52,091,058	19.44	17.66	21.4	19.19
14	REBIF	361	139,379	37,420	51,982,161	3.6	3.24	3.99	3.59
15	STELARA	255	146,816	37,526	51,974,724	2.41	2.13	2.72	2.4
16	SANDOSTATIN LAR DEPOT	247	105,871	37,534	52,015,669	3.23	2.85	3.66	3.23
17	NEXIUM	239	231,857	37,542	51,889,683	1.42	1.25	1.62	1.42
18	RINVOQ	223	104,313	37,558	52,017,227	2.96	2.6	3.38	2.96
19	XELJANZ XR	222	189,565	37,559	51,931,975	1.62	1.42	1.85	1.62
20	ELIQUIS	187	173,134	37,594	51,948,406	1.49	1.29	1.72	1.49
21	CIPROFLOXACIN	176	160,601	37,605	51,960,939	1.51	1.3	1.76	1.51
22	TRUVADA	168	75,833	37,613	52,045,707	3.07	2.63	3.57	3.06
23	VIREAD	148	103,580	37,633	52,017,960	1.98	1.68	2.32	1.97
24	AUBAGIO	128	112,094	37,653	52,009,446	1.58	1.33	1.88	1.58
25	TRULICITY	124	137,806	37,657	51,983,734	1.24	1.04	1.48	1.24
26	XYWAV	118	21,481	37,663	52,100,059	7.6	6.34	9.11	7.56
27	ALLI	96	45,753	37,685	52,075,787	2.9	2.37	3.54	2.9
28	NATPARA	81	9,709	37,700	52,111,831	11.53	9.26	14.35	11.44
29	ATRIPLA	74	41,754	37,707	52,079,786	2.45	1.95	3.08	2.45
30	GLIVEC	73	47,590	37,708	52,073,950	2.12	1.68	2.67	2.12

**FIGURE 3:**
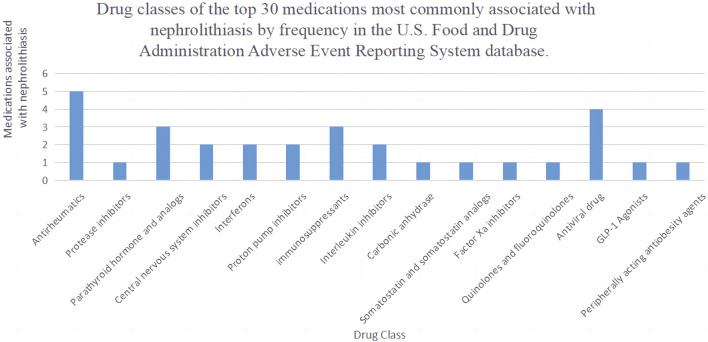
Drug classes of the top 30 medications most commonly associated with nephrolithiasis by frequency.

The list of drugs includes a variety of antiretroviral drugs, proton pump inhibitors, immunomodulators, tumor necrosis factor (TNF) blockers, anticoagulants, parathyroid hormone-related drugs (ATAZANAVIR, TRUVADA, VIREAD, ATRIPLA, PREVACID, NEXIUM, AUBAGIO, STELARA, ELIQUIS, NATPARA, FORTEO, COSENTYX, VEDOLIZUMAB). Other drugs include central nervous system depressants (XYREM, XYWAV), rheumatoid arthritis medications (HUMIRA, ENBREL, REMICADE, RINVOQ, XELJANZ XR), antiepileptic drugs (TOPAMAX), lipid-lowering drugs (ALLI), somatostatin analogs (SANDOSTATIN LAR DEPOT), antidiabetic drugs (TRULICITY), anticancer drugs (REVLIMID), antibiotics (CIPROFLOXACIN), protein kinase inhibitors (GLIVEC), and beta interferon-like drugs (AVONEX, REBIF). As shown in Figure 3. Among the top 30 drugs, we found that the most common drug classes associated with the occurrence of kidney stones are Antirheumatics, Antiviral drugs, parathyroid hormone and analogs, and Immunosuppressants.

In the subgroup analysis of drugs, the three most common drugs that cause kidney stones are Antirheumatics (ROR 2.1, 95% CI 2.06–2.14, PRR 2.1), Parathyroid hormone and analogs (ROR 2.67, 95% CI 2.58–2.76, PRR 2.66), and Antiviral drugs (ROR 2.48, 95% CI 2.39–2.58, PRR 2.48), as shown in Table 5. To further delve into the differences within these three drug subgroups, we conducted a differential analysis of drug categories, as seen in Table 6. Parathyroid hormone and analogs, as well as Antiviral drugs, were found to be more likely to cause kidney stones compared to Antirheumatics.

**TABLE 5:** Report-odds ratio (ROR) and Proportional Report-ratio (PRR) analysis of the top three most common drug classes associated with kidney stone formation in the FDA FAERS database from 1 January 2010 to 31 March 2024.

Ranking	Drug classification	a	b	c	d	ROR	ROR lower bound, 95% CI	ROR upper bound, 95% CI	PRR
1	Antirheumatics	17,698	15,404,821	20,083	36,716,719	2.1	2.06	2.14	2.1
2	Parathyroid hormone and analogs	3,678	2,026,199	34,103	50,095,341	2.67	2.58	2.76	2.66
3	Antiviral drug	2,890	1,685,034	34,891	50,436,506	2.48	2.39	2.58	2.48

**TABLE 6:** Report-odds ratio (ROR) and proportional report-ratio (PRR) analysis of the top three most common drug groups associated with kidney stone formation in the FDA FAERS database from 1 January 2010 to 31 March 2024.

Ranking	Drug classification	a	b	c	d	ROR	ROR lower bound, 95% CI	ROR upper bound, 95% CI	PRR
1	Parathyroid hormone and analogs:Antirheumatics	3,678	2,026,199	17,698	15,404,821	1.58	1.52	1.64	1.58
2	Antiviral drug:Antirheumatics	2,890	1,685,034	17,698	15,404,821	1.50	1.44	1.55	1.49
3	Parathyroid hormone and analogs: Antiviral drug	3,678	2,026,199	2,890	1,685,034	1.06	1.01	1.11	1.06

Among patients reporting adverse events of nephrolithiasis, The male to female ratio was 1:1.34. The age distribution was mainly concentrated in the age group of 18–65 years (42.6%). The majority of reporters were consumers (51.4%), and the countries reporting the most were the United States (52.1%) and Canada (13.1%). Detailed demographic information is provided in Table 7.

**TABLE 7:** Basic information on adverse event (nephrolithiasis) reports.

Characteristics	Case number, n	Case proportion, %
Number of events	37,027	
Gender		
Male	14,700	39.7
Female	19,701	53.2
Unknown	2,626	7.1
Age		
<18	564	1.5
18≥and≤65	15,778	42.6
>65	6,844	17.7
Unknown	14,147	38.2
Reported Person		
Consumer	19,029	51.4
Physician	7,203	19.5
Pharmacist	1,225	3.3
Registered nurse	19	0.1
Lawyer	740	2.0
Healthcare professional	3,576	9.7
Other health-professional	3,504	9.5
Unknown	1731	4.7
Reported Countries (Top two)		
America	19,284	52.1
Canada	4,858	13.1

To comprehensively study the outcomes of patients experiencing nephrolithiasis, the study conducted a detailed analysis of patient trajectories, with specific results available in Table 8. Among patients reporting adverse reactions of nephrolithiasis, the proportion of deaths was 2.6%. Additionally, over 42% of patients reported events leading to hospitalization or prolongation of hospital stay, indicating that nephrolithiasis may have a significant impact on patient health status and treatment processes.

**TABLE 8:** Patient regression of adverse event reports.

Patient outcomes		
Characteristics	Case number, n	Case proportion, %
Hospitalization	15,547	42.0
Death	971	2.6
Life-Threatening	615	1.7
Disability	271	0.7
Congenital malformation	27	0.1
Permanent defect	46	0.1
Other Outcome	14,670	39.6
Unknown	4,880	13.2

## 4 Discussion

The earlier studies indicated that sulfa drugs were the first to cause kidney stones to form. The metabolites of sulfonamides, which include crystallized substances, can deposit in the kidneys, leading to the development of kidney stones (R´eveillaud and Daudon, 1986; Catalano-Pons et al., 2004; Roedel et al., 2021; Rapado et al., 1987b). Over time, occasional observations have reported instances of other drugs contributing to kidney stone formation. For instance, indinavir, used for HIV infection treatment, has been noted to increase the incidence of kidney stones, while the occurrence of kidney stones due to calcium/vitamin D supplements or carbonic anhydrase inhibitors may be underreported (Servais et al., 2006).

It was not until the early 1980s that the concept of drug-induced nephrolithiasis started to gain individualization and conceptualization within the broader framework of drug-induced nephropathy (Curtis, 1979; Daudon et al., 1983; Rapado et al., 1987c). However, with the reduction in the prescription volume of some drugs, the attention to their role in causing nephrolithiasis gradually decreased. Examples include sulfonamides. Thus, the establishment of a real-time updated list of medications associated with nephrolithiasis becomes imperative. As far as we are aware, Daudon M and his team are among the most likely researchers to have established a list of medications potentially linked to nephrolithiasis (Daudon et al., 1982). Their research indicated that several drug categories, including proteinase inhibitors, antiviral drugs, potassium-sparing diuretics, sulphonamides, silicate-containing drugs, antibacterial agents, carbonic anhydrase inhibitors, calcium/vitamin D supplements, and antiepileptic drugs, have been associated with nephrolithiasis. Subsequently, the mechanisms of the link between these medications and the nephrolithiasis they induce have been studied through the analysis of stone composition.

This study aims to expand upon the research by Daudon M and his team by analyzing the 30 most frequently reported medications inducing nephrolithiasis in the FAERS. There are several reasons why the research by Daudon M et al. requires an update. Firstly, with the ongoing updates to medical guidelines and the continuous approval of new drugs, along with the gradual withdrawal of obsolete medications, the list of medications causing nephrolithiasis is rapidly evolving. Additionally, although previous studies have established a foundation for evaluating the typical nephrolithiasis associated with medications, our study employs the proportionate reporting rate (PRR) and the Reporting Odds Ratio (ROR) as the primary analytical methods, reporting the overall frequency of reporting nephrolithiasis among medications.

Naturally, one warning worth heeding is that, despite the absence of certain drug categories mentioned in the initial study, such as sulfonamides, from our top 30 list, this does not necessarily imply that these categories of drugs are no longer linked to nephrolithiasis. Instead, this observation may suggest that, in comparison, reports pertaining to various drug categories are now being made more frequently. However, as the observation of drug usage deepens, compiling a new list specifically identifying the medications that induce nephrolithiasis is undoubtedly set to assume greater significance as a reference.

In comparison to the research by Daudon M, our study has identified new drugs associated with nephrolithiasis among the top 30, such as immunomodulators (AUBAGIO), proton pump inhibitors (PREVACID, NEXIUM), and parathyroid hormone-related drugs (TERIPARATIDE, NATPARA, FORTEO). The most common 30 drugs, mechanisms of action, factors affecting kidney stones, and types of stones are listed in Supplementary Table 1.

Aubagio, an immune modulator, is employed in the management of multiple sclerosis, effectively inhibiting the activity of mitochondrial enzymes in a selective and reversible fashion. Current research indicates that the suppression of urate reabsorption by AUBAGIO contributes to the development of renal calculi. There is evidence of an augmented risk of uric acid nephrolithiasis in individuals with multiple sclerosis during AUBAGIO therapy. It is recommended that patients take alkalizing agents to lessen the occurrence of renal stones (Largeau et al., 2021).

Hormonal therapies, including those simulating the action of parathyroid hormones,are used to treat parathyroid gland dysfunctions. Primary hyperparathyroidism is a disease caused by excessive secretion of parathyroid hormone (PTH) by the parathyroid glands (Zanocco and Yeh, 2017). Both scenarios lead to elevated levels of PTH in the blood. The hallmark of primary hyperparathyroidism is hypercalcemia, a condition characterized by elevated levels of calcium in the blood (Cong et al., 2018). One of its most common complications is the formation of kidney stones (Zhou et al., 2022; Vestergaard, 2015). Numerous studies have indicated that increased secretion of parathyroid hormone raises the concentration of calcium ions in the blood and promotes the excretion of calcium in the urine, leading to increased levels of calcium in urine. When the concentration of calcium ions in urine reaches saturation, excess calcium ions can crystallize if there is hypercalciuria (Zhou et al., 2022; Vestergaard, 2015; Broadus, 1982). In addition to hypercalciuria, low magnesium in urine and an elevated calcium-to-magnesium ratio in urine are also associated with kidney stones (Saponaro et al., 2020). However, Forteo can increase blood calcium levels, leading to hypercalciuria, potentially contributing to the formation of calcium kidney stones. It can also disrupt the balance of calcium and phosphate in the urine, creating favorable conditions for stone formation. Unlike primary hyperparathyroidism, Teriparatide causes intermittent rather than sustained increases in urinary calcium, thus the risk of kidney stones should be lower or non-existent. Further research is needed to fully understand the mechanisms by which it contributes to the formation of kidney stones.

Proton pump inhibitors (PPIs), a globally prevalent prescription medication for treating conditions related to stomach acid, have been the subject of significant concern due to their overuse (Savarino et al., 2018). Studies have shown a positive correlation between the use of PPIs over an extended period and the incidence and recurrence rates of kidney stones (Liu et al., 2023; Sui et al., 2022). This may be attributed to the inhibition of gastric acid secretion and reduced intestinal absorption, leading to hypomagnesemia and hypocitraturia, which in turn increases the risk of kidney stones (Sui et al., 2022; Simonov et al., 2021; William et al., 2014). These mechanisms may serve as important considerations in the close association between PPIs and kidney stones. Consequently, patients who have been using proton pump inhibitors (PPIs) for an extended period are advised to pay special attention to the risk of kidney stones.

The occurrence of renal stones is a significant issue that has been documented in association with the use of certain medications, and among these, antiretroviral drugs (atazanavir, truvada, atripla, viread) and antiepileptic drugs (topamax) stand out as particularly notable.

Atazanavir, an antiretroviral medication belonging to the category of HIV protease inhibitors, is used in the treatment of human immunodeficiency virus (HIV) infection. Due to its single daily dose, low capsule load, and high antiviral activity, the frequency of use is gradually increasing. (Nishijima et al., 2013). Atazanavir is an antiretroviral drug belonging to the category of HIV protease inhibitors, used in the treatment of human immunodeficiency virus (HIV) infection. Due to its daily single-dose administration, low capsule burden, and high antiviral activity, its use is becoming increasingly common (Nishijima et al., 2013). In this study, atazanavir was reported to be associated with 702 cases of nephrolithiasis, with a risk of recurrent occurrence (ROR) of 46.35 (95% CI: 43–50), indicating a very high risk of developing nephrolithiasis. In contrast, the number of reports for similar drugs is relatively small, with darunavir at 10 cases and indinavir at 5 cases. This may be due to the fact that atazanavir is increasingly replacing other antiretroviral drugs in clinical use, leading to an increase in its use and, consequently, the risk of developing nephrolithiasis. Several studies have shown an increased risk of nephrolithiasis among patients treated with atazanavir (Tattevin et al., 2013; Chan-Tack et al., 2007; Rockwood et al., 2011; Hamada et al., 2012; Valencia and Moreno, 2009). The incidence of stones in the atazanavir group was approximately 10.8 times that of antiretroviral drugs (Chan-Tack et al., 2007), with a median time from starting treatment to diagnosis of nephrolithiasis of 2.2 years (Rockwood et al., 2011). Among the 13 patients who discontinued atazanavir, there were no recurrences of stones, while among the 18 patients who continued treatment, a third experienced recurrent stones (Hamada et al., 2012). There have also been multiple reports of nephrolithiasis in HIV-infected individuals receiving antiretroviral therapy (including atazanavir) (Valencia and Moreno, 2009; Wang et al., 2014; Plawecki et al., 2023). The mechanism by which atazanavir causes nephrolithiasis is primarily related to its low solubility (Couzigou et al., 2007). Analysis using FTIR (Fourier Transform Infrared) spectroscopy determined that atazanavir was the primary component in the kidney stones induced by the drug (Valencia and Moreno, 2009). However, Atazanavir can be improved by preparing arsenic-loaded nanoparticles (ASNPs) to improve its water solubility. Solubility enhancement could be attributed to the decrease in crystallinity of Atazanavir when dispersed in NPs (Dhabliya et al., 2022).In summary, for HIV patients taking atazanavir, it is important to be highly vigilant about the possibility of drug-induced nephrolithiasis.

Topamax, an antiepileptic drug, is used in the treatment of epilepsy. It achieves its effect by blocking the voltage-gated sodium and calcium channels, inhibiting glutamate receptors, enhancing GABA (gamma-aminobutyric acid) receptors, and inhibiting carbonic anhydrase activity, thereby preventing seizures. (Pearl et al., 2023). Reports of renal stones associated with topamax use have been documented in both adult and pediatric populations (Alarcón-Martínez et al., 2006; Merino-Salas et al., 2014; Fukumoto et al., 2011).The risk of developing renal stones while taking topamax is approximately 2–4 times higher than that expected in the general population (Lamb et al., 2004). Research has indicated a strong correlation between topamax use and several conditions that contribute to the formation of renal stones, including metabolic acidosis, hypokalemia, high urine pH levels, hyperuricosuria, and low citrate uraturia. These factors, in turn, predispose patients to the formation of renal stones. The use of topamax in children for long-term epileptic treatment has been linked to persistent hypercalciuria and metabolic acidosis, which can lead to renal calciphylaxis or stone formation (Dell'Orto et al., 2014; Maalouf et al., 2011; Gupta et al., 1995). Furthermore, in children with severe disabilities who suffer from epilepsy (Barnett et al., 2018), the incidence of kidney stones or calcifications is significantly higher in the group treated with topamax (60% vs. 0%, p = 0.00241) (Ishikawa et al., 2019).

Furthermore, in our list, we also discovered several medications that may be associated with the occurrence of renal stones.

Ciprofloxacin is a fluoroquinolone antibiotic (Pearl et al., 2023; Alarcón-Martínez et al., 2006; Merino-Salas et al., 2014; Fukumoto et al., 2011; Lamb et al., 2004; Dell'Orto et al., 2014; Maalouf et al., 2011; Gupta et al., 1995; Barnett et al., 2018; Ishikawa et al., 2019; Sica and Gehr, 1989; Bhatt and Chatterjee, 2022), which, unlike other fluoroquinolones, only causes crystalluria at high doses and when urine pH is alkaline. (Thorsteinsson et al., 1986; Morell-Garcia et al., 2015). However, there have been documented instances of ciprofloxacin-induced stone formation and bilateral hydronephrosis (Chopra et al., 2000). This may be related to the ciprofloxacin crystal formation caused by the low solubility of high-dose ciprofloxacin and the excretion of approximately 40%–50% of the drug in its original form, leading to interstitial nephritis of the renal tubules and acute renal failure symptoms (Lomaestro, 2000; Vissers et al., 2022). Additionally, changes in the intestinal microbiota may also be involved in this phenomenon (Stern et al., 2021).

Research has shown that factors associated with drug-induced kidney stones include low solubility of the drug, high doses of the drug, prolonged treatment duration, and the drug’s impact on altering the pH of urine (Sighinolfi et al., 2019). The low solubility of the medication is a major cause of medication-induced kidney stone formation. For example, nevirapine may crystallize in the urine and form kidney stones due to its low solubility. In addition, changes in urinary pH are also important for kidney stone formation. (Rodgers, 2017). Uric acid crystallizes in urine with a pH less than 5.5, forming insoluble, undissociated uric acid crystals. Therefore, uric acid kidney stones typically occur in individuals with low urine pH but normal uric acid levels. (Liebman et al., 2007). In contrast, calcium phosphate crystallizes into hydroxyapatite at urine pH greater than 6.0, and the formation of stones is further facilitated by factors such as dehydration by crystallization inhibitors. (Grases et al., 2002). Calcium oxalate crystals form independently of urinary pH, as the solubility of monohydrated or dihydrated calcium oxalate does not change significantly at physiological urinary pH (Finlayson, 1978). The presence of heteronuclei is a necessary condition for the formation of calcium oxalate crystals (Eisner and Goldfarb, 2014). Uric acid and hydroxyapatite crystals have been shown to serve as heteronuclei for the formation of calcium oxalate crystals, as uric acid ions and phosphate ions can promote heterogeneous nucleation and enhance the attachment of crystals to epithelial cells (Aggarwal et al., 2013; Carbone et al., 2018). A urinary pH of <5.5 or >6.0 can respectively induce the crystallization of uric acid or calcium phosphate, potentially leading to the formation of calcium oxalate kidney stones under appropriate conditions. Studies have shown that low pH urine can cause the crystallization and precipitation of CaOx crystals (Moe et al., 2002), and alkaline urine may also promote the precipitation and nucleation of CaOx crystals (Shekarriz and Stoller, 2002; Song et al., 2000). For example, AUBAGIO can lower the urinary pH, leading to the formation of uric acid crystals and the occurrence of uric acid stones. Under appropriate conditions, uric acid crystals may form heteronuclei with the metabolites of the drug to form calcium oxalate kidney stones.

Interestingly, on our list, we did not find certain medications previously recognized as contributors to kidney stones, such as ceftriaxone (Azarkar et al., 2018; Wang et al., 2017), sulfonamides (Daudon et al., 2018b; Sighinolfi et al., 2019; Antopol and Robinson, 1939; Barnes and Kawaichi, 1943; Abaza, 1946), and indinavir (Gentle et al., 1997; Plosker and Noble, 1999). With the emergence of newer medications, they are more likely to be accepted by clinical practitioners due to their advantages over previously used drugs, which in turn increases their usage. For example, nevirapine, with its once-daily single dose, low capsule burden, and high antiviral activity, has replaced indinavir as the number one medication on our kidney stone risk ranking; it has several advantages over indinavir, leading to its popularity and thus contributing to a new shift in the prevalence of medication-induced kidney stones (Broadus, 1982).

## 5 Limitations

The Reporting Odds Ratio (ROR) represents a standard method for assessing significance within pharmacovigilance databases, such as those utilized to report adverse drug reactions (Sakaeda et al., 2013). However, the calculation of ROR may inadvertently favor medications with a lower overall reporting frequency. Our current methodology endeavors to address this by ranking medications by their frequency of occurrence in the FAERS database prior to determining ROR.

A limitation of this study is the inherent biases in reporting and the FAERS database itself. Due to the nature of adverse event reporting, the database tends to receive reports of more severe and easily attributable adverse events to a specific medication. This means that less severe adverse events and those without a clear causal relationship are more likely not to be reported. Moreover, the study is limited by the FAERS database and may suffer from biases in population representation, underreporting, information gaps, and a lack of severity ratings for adverse reactions.

Furthermore, the population in this study predominantly consists of Americans and Japanese, and the occurrence of kidney stones can be related to racial demographics (Stamatelou and Goldfarb, 2023). There is a lack of real-world research from China.

It is important to note that the findings of the ROR analysis are indicative of statistical associations and cannot conclusively infer causal relationships.

Our list reports three tumor necrosis factor (TNF) inhibitors (HUMIRA, ENBREL, and REMICADE), two Janus kinase (JAK) inhibitors (RINVOQ, XELJANZ XR), two immune system modulators (COSENTYX, VEDOLIZUMAB), a lipid-lowering drug (ALLI), and a diabetes-lowering drug (TRULICITY). The indications for these medications include rheumatoid arthritis, psoriasis, Crohn’s disease, ankylosing spondylitis, and ulcerative colitis. For instance, Vedolizumab can be used to control inflammation and alleviate symptoms of inflammatory bowel diseases such as Crohn’s disease and ulcerative colitis (Rakowsky et al., 2022). Research has shown that patients receiving Vedolizumab treatment have an increased risk of kidney stones compared to those not treated, with an OR of 1.307 (95% CI 1.076–1.588, p = 0.0071) (Sakaeda et al., 2013; Alameddine et al., 2023). Currently, the potential mechanisms by which Vedolizumab induces kidney stones are not clear, and further study is required. However, some medications appear to cause kidney stones, and in reality, the actual causal relationship may be confounded by other factors. This is a general limitation associated with the use of large self-reporting pharmacovigilance databases, where adverse drug reaction associations that may be causal can only be reported. For example, inflammatory bowel diseases (such as Crohn’s disease and ulcerative colitis) increase the risk of kidney stones in patients receiving Vedolizumab therapy. However, inflammatory bowel diseases themselves are also associated with kidney stones (Kim and Jung, 2019; Zhang et al., 2023). Similarly, hyperlipidemia, diabetes, and other conditions are independent risk factors for kidney stones (Tastemur et al., 2022; Rezaee et al., 2017; Khan et al., 2016), closely related to the occurrence of kidney stones. Therefore, we cannot ascertain whether the aforementioned medications are listed due to the underlying disease itself or secondarily to the medication.

## 6 Conclusion

Our study identified the most commonly associated medications with drug-induced nephrolithiasis and their respective Reporting Odds Ratios (ROR). Our list may assist in informing clinicians of which medications should be considered in the secondary causes of nephrolithiasis or in the care of patients predisposed to nephrolithiasis. Specifically, this information may be most beneficial for patients with peptic ulcer disease and duodenal ulcers, hyperparathyroidism, epilepsy, human immunodeficiency virus (HIV) infection, and drug-induced nephrolithiasis. Further research, including prospective observational pharmacologic epidemiological studies, will help to quantify the risk of these medications causing nephrolithiasis.

## Data Availability

The datasets presented in this study can be found in online repositories. The names of the repository/repositories and accession number(s) can be found below: https://fis.fda.gov/extensions/FPD-QDE-FAERS/FPD-QDE-FAERS.html.

## Abbreviations

FAERS, the United States Food and Drug Administration’s Adverse Event Reporting System; RORs, The Reporting Odds Ratios; HIV, human immunodeficiency virus; AEs, the adverse events; CI, a 95% confidence interval; PPIs, Proton pump inhibitors.
