# Supplementary material for: An exploratory study evaluated the 30 most commonly reported medications in the United States food and drug administration’s adverse event reporting system that are associated with the occurrence of kidney stones
Source: Front Pharmacol. 2024 Dec 18;15:1377679. doi: 10.3389/fphar.2024.1377679 (PMC11701592; doi:10.3389/fphar.2024.1377679)
Supplement: Supplementary file 1 [file Table1.docx]

**Supplementary Table 1:Risk Factors and Mechanisms of Kidney Stone Formation Associated with Commonly Drugs in the FDA's FAERS Database from January 1, 2010, to March 31, 2024**

| **ranking** | **Medication** | **mechanism of action** | **Factors Increasing Stone Formation** | **Stone Composition** |
| --- | --- | --- | --- | --- |
| 1 | ATAZANAVIR | Atazanavir is a novel azapeptide protease inhibitor with high specificity for, and activity against, HIV-1 protease. | Atazanavir has been linked to the development of kidney stones, primarily because of its low solubility at physiological pH, which can result in crystallization within the urinary tract. Individuals with pre-existing kidney or liver conditions may experience a slower metabolism of atazanavir, leading to elevated concentrations in the urine and subsequently heightening the risk of stone formation. | be composed of 37%–65% unmetabolized atazanavir by weight. |
| 2 | FORTEO | Forteo is a man-made form of parathyroid hormone that exists naturally in the body. Teriparatide increases bone mineral density and bone strength, which may prevent fractures. | Teriparatide elevates blood calcium levels, leading to hypercalciuria, which can contribute to the formation of calcium-based kidney stones. Furthermore, its promotion of phosphate excretion disrupts the balance of calcium and phosphate in the urine, creating stone-forming conditions. Unlike primary hyperparathyroidism, teriparatide causes intermittent rather than sustained increases in urinary calcium. | Calcium Oxalate,  Calcium Phosphate. |
| 3 | XYREM | Xyrem, the sodium salt of gamma-hydroxybutyrate (GHB), acts as a neurotransmitter and neuromodulator in the central nervous system, primarily targeting GABA_B receptors. Activation of GABA_B receptors in the kidney can impact the diameter of renal microvessels, thereby influencing blood flow crucial for maintaining proper kidney function and adequate filtration. | Xyrem, as the sodium salt of gamma-hydroxybutyrate (GHB), can result in elevated urinary excretion of calcium, leading to the supersaturation of urine with calcium salts. This increased calcium concentration in the urine can contribute to the formation of calcium-based kidney stones, highlighting a potential mechanism for stone development. | Calcium Oxalate |
| 4 | TERIPARATIDE | Teriparatide, a parathyroid hormone analog used to treat osteoporosis resulting from menopause, steroid use, or gonadal failure, is indicated when there is a high risk of bone fracture due to osteoporosis. | Teriparatide enhances the reabsorption of calcium in the renal tubules, leading to elevated serum calcium levels. This increase in blood calcium levels can result in higher calcium excretion in the urine (hypercalciuria), potentially contributing to the formation of calcium-based kidney stones. | Calcium Oxalate |
| 5 | AVONEX | Avonex, derived from human proteins, contains interferons that aid the body in combating viral infections. This medication is specifically utilized to manage relapsing multiple sclerosis (MS) in adults, encompassing clinically isolated syndrome, relapsing-remitting disease, and active secondary progressive disease. While Avonex does not offer a cure for MS, it effectively reduces the frequency of relapse symptoms. | Interferon therapy, such as AVONEX, has been linked to nephrotoxicity, impacting renal calcium and mineral handling, thereby potentially facilitating stone formation. This treatment can elevate urinary calcium levels (hypercalciuria), a key risk factor for calcium-based kidney stone development. | Calcium oxalate, Uric acid |
| 6 | PREVACID | Proton pump inhibitors，Its mechanism of action is to selectively inhibit the membrane enzyme H+/K+ ATPase in gastric parietal cells. | PREVACID could result from the inhibition of gastric acid secretion and decreased intestinal absorption, causing hypomagnesemia and hypocitraturia, ultimately elevating the risk of kidney stone formation. | Calcium oxalate |
| 7 | REVLIMID | Revlimid, a medication prescribed for various cancers and anemia in adults, functions by slowing cancer growth, inhibiting new blood vessel formation in tumors, and enhancing the immune system's ability to combat cancer . | Revlimid treatment can affect how the body handles calcium, leading to increased levels of calcium in the urine, known as hypercalciuria. This condition poses a significant risk for the formation of kidney stones composed of calcium. | Calcium oxalate |
| 8 | COSENTYX | It is a selective interleukin-17A (IL-17A) inhibitor that blocks the binding of IL-17A to its receptor, thereby disrupting the signaling pathway of the inflammatory response and reducing inflammation triggered by the immune system. | Inhibition of IL-17A may lead to changes in the body's metabolism and mineral balance, increasing the concentration of calcium in the urine. | Probably Calcium oxalate |
| 9 | VEDOLIZUMAB | Vedolizumab targets the α4β7 integrin on gut-homing T-lymphocytes, essential for their migration into the gastrointestinal tract. As an integrin receptor antagonist, it inhibits gut lymphocyte trafficking. | Inflammatory bowel diseases (IBD), which Vedolizumab treats, often impair nutrient absorption. Reduced absorption of magnesium, a natural inhibitor of kidney stones, may increase the risk of stone formation. | Probably Calcium oxalate |
| 10 | TOPAMAX | Topiramate is a carbonic anhydrase inhibitor, which blocks the enzyme crucial for bicarbonate reabsorption in the kidneys. This inhibition causes a decrease in bicarbonate reabsorption, leading to type II (proximal) renal tubular acidosis, characterized by a normal anion gap metabolic acidosis. | Topiramate, a medication known to reduce citrate levels in urine, can potentially increase the risk of calcium phosphate stone formation. This is due to the inhibition of renal proximal tubule carbonic anhydrases by TPM, leading to systemic acidosis and a decrease in bicarbonate reabsorption. As a result, the final urine pH increases, which reduces the binding of citrate to calcium, making it more available for stone formation. | calcium phosphate |
| 11 | SANDOSTATIN LAR DEPOT | Sandostatin LAR Depot is a long-acting formulation of octreotide, a somatostatin analogue used to treat diarrhea and flushing caused by cancer, as well as acromegaly. | The enteric hyperoxalosis, oxaluria and urolithiasis were presumably side effects of the Octreotide treatment. | The calculi consisted of oxalatet |
| 12 | NEXIUM | Nexium is a proton pump inhibitor that works by covalently binding to the sulfhydryl groups of cysteines on the H+/K+ ATPase enzyme, also known as the proton pump, located on the secretory surface of gastric parietal cells. This binding inhibits the final step in gastric acid production, leading to a significant reduction in both basal and stimulated gastric acid secretion. | Nexium may reduce calcium absorption in the intestines, leading to compensatory hypercalciuria. | Calcium oxalate |
| 13 | ELIQUIS | Eliquis selectively inhibits Factor Xa, directly binding to Factor Xa to block the conversion of prothrombin to thrombin. | Eliquis may potentially increase the risk of kidney stone formation by inhibiting Factor Xa, which can lead to reduced urine flow due to conditions requiring anticoagulation, such as prolonged immobility after surgery or illness. | Not clear yet |
| 14 | CIPROFLOXACIN | Ciprofloxacin is one of a new generation of fluorinated quinolones structurally related to nalidixic acid. The primary mechanism of action of ciprofloxacin is inhibition of bacterial DNA gyrase. | When used in large doses, its metabolites have low solubility in urine and may lead to the formation of drug crystals in urine, especially in alkaline urine. Such crystals may be deposited in the kidneys, forming stones. | Ciprofloxacin |
| 15 | TRUVADA | Contains two active ingredients: Emtricitabine and Tenofovir.The former is a nucleoside reverse transcriptase inhibitor (NRTI) that inhibits viral replication by interfering with the reverse transcription process of HIV.The latter is also an NRTI that competitively inhibits HIV reverse transcriptase and inserts into the viral DNA chain, thereby terminating the extension of viral DNA and preventing HIV replication. | Possibly due to tubular damage caused by it, leading to proximal tubulopathy (Fanconi syndrome), which affects the abnormal renal handling of calcium, phosphate, and other electrolytes, ultimately resulting in the formation of kidney stones. | Calcium phosphate |
| 16 | VIREAD | Viread is the first nucleotide analog reverse transcriptase inhibitor to be approved by the Food and Drug Administration for the treatment of HIV infection. | Similar to Travada | Calcium phosphate |
| 17 | AUBAGIO | Aubagio works by reducing lymphoid cell proliferation through the inhibition of dihydroorotate dehydrogenase, an enzyme involved in de novo pyrimidine synthesis. | Aubagio has uric acid excretion effect. This means that it may increase the concentration of uric acid in the urine by increasing the way uric acid is excreted from the body, which may lead to the formation of uric acid stones. | Uric acid stones |
| 18 | TRULICITY | Trulicity stimulates insulin secretion by activating the glucagon-like peptide-1 (GLP-1) receptor, inhibits the release of glucagon, delays gastric emptying, and helps control blood sugar levels by reducing appetite. | Trulicity can slow gastric emptying and suppress appetite, and some people may eat less, especially water. Inadequate water intake can cause urine to become concentrated, increasing the concentration of minerals (such as calcium, oxalate, and uric acid) in the urine, which can increase the risk of stones. | Calcium oxalate |
| 19 | XYWAV | Xywav contains an active ingredient that is a metabolite of gamma-aminobutyric acid (GABA). This component helps regulate sleep cycles by controlling the inhibitory neurotransmitter GABA in the brain. | Sodium oxalate in medications may increase the body's oxalate load. High oxalate intake increases the concentration of oxalate in urine, further increasing the risk of calcium oxalate stone formation. | Calcium oxalate |
| 20 | ALLI | Alli mainly inhibits the activity of pancreatic lipase and gastric lipase, preventing the decomposition of dietary fat and making it impossible for fat to be absorbed by the intestines. | Unabsorbed fat and bile acids may react with calcium in the intestinal lumen, limiting the amount of free calcium binding with oxalate and thereby raising intestinal oxalate absorption leading to hyperoxaluria. | Calcium oxalate |
| 21 | NATPARA | Natpara is a synthetic parathyroid hormone that regulates calcium and phosphorus metabolism by mimicking the effects of the parathyroid hormone produced naturally by the body. | While helping to maintain normal blood calcium levels, long-term use may result in calcium deposits in the kidneys, especially if blood calcium is not properly controlled. |  |
| 22 | ATRIPLA | An antiretroviral drug used to treat HIV infection. | Similar to Travada | Calcium phosphate |
| 23 | GLIVEC | Glivec inhibits tumor proliferation and growth by inhibiting specific overactive tyrosine kinases (such as BCR-ABL, c-KIT, PDGFR) in certain cancer cells. | Drugs may cause renal toxicity, including tubular damage and interstitial nephritis, which reduces the ability of urine to concentrate, thereby increasing the concentration of minerals in urine and promoting crystal deposition and stone formation. | Calcium oxalate |
| 24 | HUMIRA | Humira, a tumor necrosis factor (TNF) blocker, works by reducing the effects of a substance in the body that can cause inflammation. It is utilized in the treatment of various inflammatory conditions in adults, including rheumatoid arthritis, psoriatic arthritis, ankylosing spondylitis, plaque psoriasis, and hidradenitis suppurativa. Additionally, Humira is employed in both adults and children for conditions such as Crohn's disease, juvenile idiopathic arthritis, ulcerative colitis, and uveitis. | Humira is used to treat inflammatory conditions such as rheumatoid arthritis, psoriatic arthritis, ankylosing spondylitis, plaque psoriasis, and other related conditions. The FAERS database is a large self-reporting drug surveillance database with inherent limitations, as it can only report potential causal relationships between drugs and adverse reactions. For instance, inflammatory conditions like rheumatoid arthritis and psoriatic arthritis themselves can lead to the occurrence of kidney stones. Therefore, we cannot definitively attribute adverse reactions to the drug alone, as they may also be related to the underlying conditions being treated. | Not clear yet |
| 25 | ENBREL | Enbrel, a tumor necrosis factor (TNF) blocker, functions by reducing the levels of TNF. It is indicated for the treatment of rheumatoid arthritis, psoriatic arthritis, and ankylosing spondylitis to prevent joint damage. | Similar to Humira | Not clear yet |
| 26 | REMICADE | Remicade functions by reducing the effects of a substance in the body that triggers inflammation. It is employed in the treatment of various conditions, including rheumatoid arthritis, psoriatic arthritis, ankylosing spondylitis, and severe or disabling plaque psoriasis in adults. Additionally, Remicade is utilized to manage ulcerative colitis and Crohn's disease in both adults and children aged at least 6 years old. | Similar to Humira | Not clear yet |
| 27 | REBIF | Interferon beta-1a for multiple sclerosis works by binding to cell surface receptors, activating Jak1 and Tyk2 kinases. These kinases phosphorylate STAT proteins, leading to gene expression changes that can produce pro-inflammatory cytokines and other inflammatory mediators. | Interferon therapy may increase the risk of uric acid nephrolithiasis by inducing hyperuricemia. | Uric acid stones |
| 28 | STELARA | Stelara blocks proteins called IL-12 and IL-23, which cause inflammation in autoimmune conditions like plaque psoriasis, psoriatic arthritis, Crohn’s disease, and ulcerative colitis. By inhibiting these proteins, Stelara reduces inflammation, pain, swelling, and skin symptoms associated with these conditions. | Similar to Humira | Not clear yet |
| 29 | RINVOQ | Rinvoq is an oral Janus kinase (JAK) inhibitor that treats inflammatory conditions by reducing immune system activity. It is indicated for rheumatoid arthritis, psoriatic arthritis, eczema, ulcerative colitis, and Crohn's disease. | JAK inhibitors increase the risk of infections, suppress the immune system, and may lead to the occurrence of kidney stones. However, due to their indications for rheumatoid arthritis, psoriatic arthritis, ulcerative colitis, and Crohn's disease, similar situations to Humira may arise. It is not yet clear whether the occurrence of kidney stones is due to the diseases themselves or the use of the medication. | Not clear yet |
| 30 | XELJANZ XR | Xeljanz XR is a Janus kinase (JAK) inhibitor that works by blocking the action of specific enzymes in the body that are involved in the inflammation process. This inhibition helps to reduce the immune response that causes inflammation in conditions such as rheumatoid arthritis, psoriatic arthritis, and ulcerative colitis. | Similar to Rinvoq | Not clear yet |
